# Editorial: Advanced Therapies for Cardiac Regeneration

**DOI:** 10.3389/fbioe.2021.644076

**Published:** 2021-03-04

**Authors:** Monica Boffito, Susanna Sartori, Ipsita Roy, Irene Carmagnola, Valeria Chiono

**Affiliations:** ^1^Department of Mechanical and Aerospace Engineering, Politecnico di Torino, Turin, Italy; ^2^Department of Materials Science and Engineering, The University of Sheffield, Sheffield, United Kingdom

**Keywords:** cardiac tissue engineering, regenerative medicine, scaffolds, biomaterials, hydrogels, stem cells, reprogramming, physico-chemical stimuli

Cardiovascular diseases (CVDs) are the leading cause of death worldwide, accounting for ~18 million deaths annually (WHO data). The term CVD gathers a group of different disorders involving the heart, its constituent structures, and the blood vessels. Among CVDs, coronary heart disease and stroke are responsible for four out of five CVD-related deaths, and one third of these deaths occur prematurely in people aged 70 or younger. The progressive or sudden obstruction of the coronary arteries is responsible for the onset of myocardial infarction which initiates a detrimental cascade of events finally leading to heart failure. More in detail, heart failure results from the continuous remodeling of the scar tissue replacing the beating heart muscle in the infarcted region, and represents a chronic condition in which the heart muscle progressively loses its ability to pump enough blood to fulfill the needs of all the body's compartments. Heart failure thus represents the main cause of morbidity and mortality of myocardial infarcted patients in the long term. Within this context, cardiac tissue engineering/regenerative medicine (TERM) strategies could arise as cutting-edge therapies in the management of myocardial infarcted patients, opening the way to the possibility to replace the damaged heart tissue and recover its functionality. Such an approach could effectively represent a valid alternative to the gold standard heart transplantation, encompassing all issues related to donor shortage and the need for life-long administration of immunosuppressive therapies. Among other common cardiovascular diseases, we also recall valve heart diseases and cardiomyopathies. It must be highlighted the strong associations existing between different cardiovascular diseases, such as coronary heart disease and valvular heart disease, cardiomyopathy and heart failure. This observation indicates that a multiple regenerative medicine approach, which considers different diseases, could be an effective strategy in the management of CVDs. The Research Topic “Advanced Therapies for Cardiac Regeneration” aims at presenting a series of articles summarizing the latest research updates on cardiac TERM approaches which combine cells, biomaterials, hydrogels, tissue engineered scaffolds/patches and physico-chemical stimuli to achieve the ultimate goal of regenerating the injured heart tissue. The issue is comprised of 19 peer-reviewed manuscripts (nine reviews, two perspectives, seven original research articles, and one Brief Research Report) derived from the many fields involved in the topic, namely (bio)materials science and engineering, biology, biotechnology, and biomedical engineering.

To better contextualize the Research Topic, the review by Montero et al. presents an in-depth overview of the specific characteristics of the myocardium that determine the needs and requirements cardiac TERM has to meet, with particular emphasis on heart tissue components, architecture and biophysical properties. The authors also briefly revise the key components required to design new cardiac TERM approaches, namely cells, materials, maturation stimuli, and scaffold fabrication techniques. Given the central role of biomaterials in the establishment of cardiac TERM therapies, Bar and Cohen focus their review on the current application of biomaterials in the field of cardiac regeneration, mainly discussing their use as forming materials for nano-carriers and matrices for cardiac regeneration induced by biomolecule release, injectable hydrogels for cell delivery, and cardiac patches. Further and more detailed insight on the use of specific biomaterials in cardiac TERM are provided by Cattelan et al. and Gonzalez De Torre et al., with particular emphasis on their application as hydrogel constituents. Being three-dimensional highly hydrated networks showing mechanical properties similar to soft tissues, hydrogels hold great promise in cardiac TERM. Cattelan et al. elucidate the promising properties of alginate in cardiac regeneration strategies and the outcomes of clinical trials in which this material has been tested to treat myocardial infarcted patients. An additional demonstration of the suitability of this material for cardiac TERM is provided by Bloise et al. who develop alginate hydrogels for the controlled release of immunomodulatory and reparative cytokines (anti-inflammatory interleukins 4/6/13 and colony-stimulating factor) to direct immune cell fate and control the wound healing process in the ischemic heart. The therapeutic ability of the proposed treatment has been proved in rat models by macrophage polarization toward healing and the improved global cardiac functionality. Differently, Gonzalez De Torre et al. discuss on the potential of elastin-based biomaterials as constituents of hydrogel scaffolds, injectable systems, or complex devices (e.g., heart valves, stents) to treat CVD-affected patients. In this regard, Fernàndez-Colino et al. investigate the use of elastin-like recombinamers as forming materials of small caliber compliant vascular grafts. The authors describe material processing into macroporous three-dimensional structures favoring cell homing, extracellular matrix (ECM) deposition and endothelium development, while exhibiting non-thrombogenicity and elastic properties mimicking the native elastin. Graft textile components are finely designed to confer proper suture retention, long-term structural stability, burst strength and compliance. The proper selection of the biomaterials used as constituents of cardiac scaffolds/patches/devices or hydrogels thus represents the first step toward the engineering of successful regenerative strategies for the management of CVD-affected patients. Indeed, biomaterials strongly affect the possibility to provide the resulting devices with proper biological signals and physical stimuli to achieve the final goal of recovering the initial organ/tissue functionality. In this regard, Belviso et al. report on the potential of decellularized human skin as scaffold to restore the native ECM which plays a pivotal role in guiding heart compliance, cardiomyocyte maturation, and function. The authors argue that a dermal matrix could effectively represent a viable alternative biological scaffold in cardiac TERM, with the key advantages of being autologous, easily accessible, and cost-effective. On the other hand, decellularized organ-derived ECM (dECM) can also be exploited to design hydrogels, thus combining the ECM characteristic pro-regenerative properties with the possibility to shape the material according to specific requirements. Liguori et al. investigate the differences among dECM hydrogels prepared starting from cardiovascular tissues of different origin, namely left ventricle, mitral valve, and aorta. The authors demonstrate that the source of dECM plays a pivotal role in determining cell differentiation and vascular network formation, because of the different molecular and biomechanical cues provided to cells. For instance, ventricular dECM hydrogels exhibit more robust vascular network formation, while aorta-derived dECM drives adipose-derived stromal cell differentiation toward a myogenic phenotype in the absence of TGF-β1 supplement to the culture medium. The readers can also find a thorough survey on the use of decellularized cardiac ECM as biomaterial for cardiac TERM in the review by Maghin et al. Besides the physical stimuli provided by the designed cell-culture platforms through their architecture and their mechanical properties, the possibility to mechanically stimulate cellularized constructs by mimicking the native milieu also promotes the development and maturation of cardiac tissue analogs, as described by Massai et al. The authors develop an automated bioreactor platform for tuneable cyclic stretch of cellularized samples coupled with a specific set-up for the *in situ* monitoring of the mechanical response of *in vitro* engineered cardiac tissues. Another key form of stimulus playing a central role in cardiac regeneration is represented by biochemical cues, as thoroughly surveyed by Cassani et al. Particularly, the authors mainly gather their attention on the crucial need of specific carriers (e.g., nanoparticles) to deliver such biochemical cues, which would ensure an efficient delivery of the therapeutic cargo, inhibit its metabolic inactivation, and avoid potential side effects. According to a different approach, proper biochemical stimuli driving cardiac repair and regeneration can be also applied by cell-secreted soluble factors. The literature review by Maghin et al. reports on the therapeutic profiling of stem cell secretome, with regard to the cardio-active ability of cell-released extracellular vesicles, including exosomes carrying cardioprotective and regenerative RNA molecules. An in-depth analysis of the potential and the mechanisms of action of miRNAs as mediators of cardioprotection is provided by Nazari-Shafti et al. The authors highlight the crucial need to identify all miRNAs involved in cardioprotection and remodeling, as well as their composition and exact mechanism of action in view of their application in clinical practice. A class of miRNAs has also been reported to drive the direct reprogramming of fibroblasts into cardiomyocyte-like cells. In the perspective of a clinical translation of this approach, Paoletti et al. test the capability of miRcombo (i.e., a combination of four microRNA mimics) to guide the direct reprogramming of adult human cardiac fibroblasts into induced cardiomyocytes, as previously observed with murine fibroblasts. Other repair and regenerative strategies currently explored in literature target different aspects of the cascade initiated by myocardial insults. For instance, Seclì et al. concentrate on the different role exerted by chaperone proteins in the healthy and injured heart. In detail, in the healthy heart chaperone proteins contribute to the regulation of myocardial physiology, while under stress conditions or during cell damage events, they are secreted by myocardial cells and induce inflammation and cardiomyocyte apoptosis. Blocking chaperone activity has been already demonstrated to produce beneficial effects on heart function in preclinical models. Hence, combining the release of specific antibodies blocking extracellular chaperones with TERM approaches may represent a future innovation to treat infarcted patients. Differently, Bigotti et al. discuss the possible exploitation of agrin as mediator of cardiac regeneration. Recently published works have demonstrated that agrin can promote heart regeneration in murine models through cardiomyocyte de-differentiation and proliferation. Moving from these data, the authors survey the currently available literature on this topic, and critically discuss on the potential clinical translation of agrin-based therapies.

Last but not least, a new chapter in the TERM field has been opened over the last two decades with the discovery of induced pluripotent stem cells (iPSCs). These cells offer the advantage of being of autologous origin, while retaining the proper characteristics of embryonic stem cells, thus completely overcoming both ethical issues and rejection risks. Mazzola and Di Pasquale provide an overview on the differentiation potential of iPSCs toward cardiomyocytes and their application in cell-based therapies, highlighting the typical drawbacks of such approaches in terms of poor cell retention and risks of occurrence of adverse effects (e.g., arrhythmias). A possible route to overcome these issues consists in combining the use of iPSCs with bioengineering technologies which can retain cells in the therapeutic target while favoring their phenotypical maturation and integration in the host tissue. iPSCs can also be successfully used as cell source in the establishment of cardiac tissue models. In addition, their autologous origin can be exploited to recapitulate *in vitro* human cardiac pathophysiology. In this regard, Jelinkova et al. report the design of an *in vitro* human model of the cardiac tissue of Duchenne muscular dystrophy (DMD)-affected patients. DMD is a severe genetic disorder associated with progressive dilated cardiomyopathy which culminates with heart failure onset, finally leading to patient death. The authors demonstrate that the designed cardiac models effectively recapitulate DMD-induced functional defects and cardiac wasting, thus offering a promising tool for a better understanding of DMD-associated cardiomyopathy and its treatment.

Finally, to complete our survey on the topic, Taghizadeh et al. exhaustively review the different types of biomaterials applied in the treatment of valvular heart diseases which represent another CVD with high incidence in developed countries, and in particular among the elderly. In the U.S. about 28,000 deaths are due to heart valve disease every year, with approx. the 60% of deaths due to disorders involving the aortic valve (data from the Centers for Disease Control and Prevention). Strict requirements in terms of material physico-chemical properties should also be fulfilled when vascular stents are engineered. Learning from a recent FDA report dealing with the adverse cardiac events and thrombosis observed at the 2-year follow-up on the first FDA-approved bioresorbable vascular stents, Pacharra et al. characterize a series of polyethylene glycol-functionalized poly-L-lactide-co-ε-caprolactone copolymers as a new generation of stent-forming materials, with improved cyto- and hemo-compatibility. As an outcome of the research work, the authors identify promising candidates for cardiovascular implant fabrication, with the additional advantage of being suitable for an easy translation of the synthesis procedure (a one-step synthesis strategy) toward industrial practice.

The reader can easily appreciate from the brief summaries of the contributions comprised in this issue that the addressed Research Topic is broad in nature and represents a still open question in the TERM field. The definition of regenerative approaches to fight the detrimental consequences of CVDs is still in its infancy. However, the cross-contamination of new ideas coming from complementary but not overlapping fields, as highlighted in the papers published within this issue, will likely lead in the future to the effective and successful repair and regeneration of injured myocardial tissue.

## Author Contributions

MB wrote the editorial, which was revised, proof-read, and approved by all authors.

## Conflict of Interest

The authors declare that the research was conducted in the absence of any commercial or financial relationships that could be construed as a potential conflict of interest.

